# Relevance of sex-differenced analyses in bioenergetics and nutritional studies

**DOI:** 10.3389/fnut.2022.936929

**Published:** 2022-09-30

**Authors:** Glòria Garrabou, Francesc Josep García-García, Rosa Elvira Presmanes, Maria Feu, Gemma Chiva-Blanch

**Affiliations:** ^1^Muscle Research and Mitochondrial Function Laboratory, Cellex-Institut d'Investigacions Biomèdiques August Pi i Sunyer (IDIBAPS), Internal Medicine Department-Hospital Clínic of Barcelona, Faculty of Medicine and Health Sciences, University of Barcelona, Barcelona, Spain; ^2^Biomedical Network Research Centre on Rare Diseases (CIBERER), Instituto de Salud Carlos III, Madrid, Spain; ^3^Department of Endocrinology and Nutrition, August Pi i Sunyer Biomedical Research Institute–IDIBAPS, Hospital Clínic of Barcelona, Barcelona, Spain; ^4^Biomedical Network Research Centre on Obesity and Nutrition Physiopathology (CIBEROBN), Instituto de Salud Carlos III, Madrid, Spain

**Keywords:** mitochondria, sex bias, nutrition, metabolism, endosymbiosis

## Abstract

Sex-biased analyses still remain as one of the biggest limitations to obtain universal conclusions. In biomedicine, the majority of experimental analyses and a significant amount of patient-derived cohort studies exclusively included males. In nutritional and molecular medicine, sex-influence is also frequently underrated, even considering maternal-inherited organelles such as mitochondria. We herein illustrate with in-house original data examples of how sex influences mitochondrial homeostasis, review these topics and highlight the consequences of biasing scientific analyses excluding females as differentiated entities from males.

## Introduction

### Role of women in history and society

The organization of mutual aid, care, empathy, and cooperation is the order of a social network that became strong and resilient for millennia of prehistory, where women sought the wellbeing of group members (1). In fact, the existence of pre-patriarchal matrilineal societies in prehistory, from the Paleolithic period of 6,500 Before the Common Era (BCE) to 3,000 BCE, is well-established by many studies in archeology. These original matrilineal societies were organized around motherhood and the offspring. Additionally, bone remains with deformities have been found in these societies, confirming that the disabled and sick were not abandoned or eliminated. These cultures were peaceful, woman-centered based on reciprocity rather than asymmetry, and in communities of up to 15,000 members (2). In this context, the construction of relations among the community members, such as symbiosis, is crucial.

Despite this lack of historic trail, materno-linearity is known as the social architecture in ancient civilizations and, still today, ranks as the preferred structure in some societies. Usually, these societies rely on cooperative networks and equitable social roles, in all life aspects including medicine. In the rest of societies, for the last 5 millennia, the paradigm of a predominantly male perspective invaded most aspects of life, including medicine and research.

The aim of this perspective is to highlight the relevance of understanding sex-specific conditions in health and disease, specifically in bioenergetics and nutritional matters, and the adverse consequences of biasing scientific and medical conclusions underscoring women's needs. In this sense, and according to the World Health Organization (WHO), sex refers to the biologically determined characteristics of females and males, whereas gender refers to the social construction as a learned behavior or identity (3). Given the biological nature of the concepts herein treated, the present work will be focused on the sex bias in relation to the study cohorts included traditionally in clinical and biomedical research, and thus human beings will be referred as males or females in this perspective.

## The endosymbiotic theory

Cooperation and networking are the aspects that have led us to move forward successfully as a species. Both aspects were highlighted, at social level, by materno-linial cultural structures as mentioned in the introduction, and at biological level, by the endosymbiotic theory postulated by Lynn Margulis (4). This theory explains the relevance of symbiotic unions in understanding the origins and the evolution of living things. The endosymbiotic theory postulates that current eucaryotic cells and other forms of life evolve together by symbiogenesis, in mutual cooperative benefit terms. Endosymbiotic theory is in contraposition to some aspects of the classical competitive Darwinian evolution, based on the survival of the fittest, and the competition to gather resources and leave genes and traits into the next generation offspring, which are currently being questioned (5, 6).

At a cellular level, symbiogenesis would explain the incorporation of plasts or mitochondria, and even a symbiogenetic origin of flagella and cilia has been suggested (undulipodia), despite there is no evidence so far (7). In the case of mitochondria, endosymbiosis is the most accepted theory to explain how we acquire mitochondrial organelles, responsible for food metabolism and energy supply. For the mitochondria, this theory postulates that the ancestral eukaryotic cell that forms our bodies engulfed, millions of years ago, an ancient proteobacterium to obtain the ability to metabolize nutrients through aerobic metabolism (thus, consuming oxygen) while providing the bacteria with food and environmental protection. Both cooperated to live together from that moment to mutually benefit from obtaining energy through the consumption of nutrients and oxygen (4).

Interestingly, mitochondria are transmitted exclusively through the maternal lineage, because during fetal conception and egg fertilization, sperm only provides half of the genetic material of the nucleus of the former embryo, while the female oocyte provides the other half of nuclear genes and all the rest of embryo components, thus including embryo mitochondria. Remarkably, mitochondria are the unique organelle of our cells that have their own genetic material that is, therefore, maternally inherited. Thus, natural selection on mitochondria operates only in females. Consequently, most of our genetic material (half the nuclear and all the mitochondrial genome) is transmitted through the maternal lineage.

Notably, the crosstalk between the nuclear and the mitochondrial genome is crucial for the cellular regulation of mtDNA integrity, copy number and, overall, mitochondrial homeostasis. Among others, because the nucleus encodes for 1,500 proteins of mitochondrial location, including those responsible of mtDNA replication and maintenance. The intergenomic communication is an additional example of bilateral cooperation regulated by many actors, including nutrients and sex-hormones (8).

## Mitochondria as an endosymbiotic evolution: A metabolic perspective

**Figure 1:**
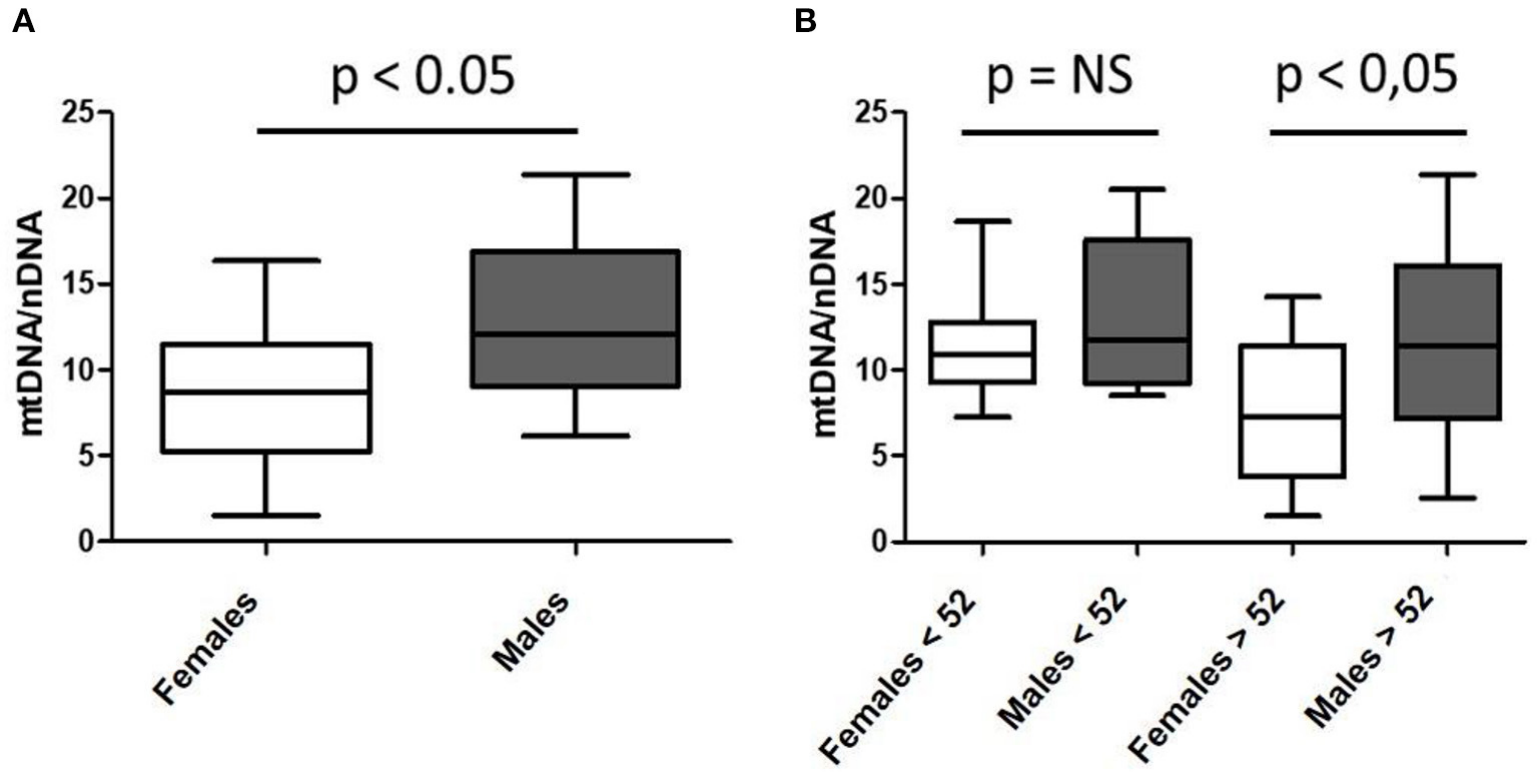
Sex-differential mitochondrial DNA (mtDNA) content in skeletal muscle of a cohort of healthy subjects according to mean age of menopause onset. **(A)** *n* = 53; 34 females and 19 males; mean age 55.94 ± 19.50 and 57.68 ± 15.06 years; **(B)** pre- and post-menopausal females (*n* = 13 and *n* = 21, respectively) and age-paired males (*n* = 8 and *n* = 11, respectively).

Despite these maternal-related mitochondrial genetic and organelle transmissions involved in nutrition and health, little interest is given in understanding sex's role in mitochondrial or nutritional pathophysiology or, in general, in females' specific biomedical needs. Accumulated knowledge stands for sex-dependent metabolism of nutrients and mitochondrial bioenergetic regulation (9–15). We herein present, as a proof of concept, novel data confirming differential sex-mitochondrial performance, in this case, related to mitochondrial DNA content (mtDNA; Figure 1). Mitochondrial DNA is present in multiple copies per mitochondria (10, 16) and, since there are thousands of mitochondria per cell, mitochondrial genome content per cell can vary from thousands to millions of copies per cell, and varies depending on the tissue considered (13). Higher mtDNA levels have been associated with more active mitochondrial function and, usually, are present in those tissues that mostly rely on oxidative metabolism, which also show a higher number of mitochondrial genomes. Conversely, low mtDNA content has been associated with disease and is usually measured for research and diagnosis of mitochondrial pathologies and associated disorders. Interestingly, their levels have rarely been associated with sex condition. We herein present original data suggesting that mtDNA levels may depend on sex assignment and that, at least in our cohort, mtDNA levels are significantly lower in the skeletal muscle of studied females (Figure 1A). When we stratify mtDNA content according to patients' age (Figure 1B), we observe that the significant differences observed in the mtDNA content between males and females, are apparently associated with the age of menopause onset (established in 52 years old) (17). This age-dependent mtDNA decline in females, although not statistically significant in this small cohort (*p* = 0.08), may be part of the metabolic reprogramming associated with physiologic aging, frailty, and disease (18).

As previously mentioned, levels of mtDNA content have been related to mitochondrial activity and disease (16). Consequently, the relevance of finding fewer mtDNA genomes in skeletal muscle of studied females, if confirmed in larger cohorts, might contribute to the understanding of differential sexual behavior in cell bioenergetics, nutrient consumption, and, eventually, health and disease. Similarly, mtDNA decrease in females according to menopause onset might confirm hormonal regulation of mitochondria or, the same way around, a potential mitochondrial contribution in menopause regulation. In this line, estrogens have been demonstrated to regulate mitochondrial function through direct and indirect mechanisms (19). In fact, they are synthetized from cholesterol in the mitochondria, together with the other steroid hormones (progestins, glucocorticoids, mineralocorticoids and androgens) (20). Consequently, estrogens are not the unique hormones related to mitochondrial function and metabolism. In fact, most of these hormones and others (such as catecholamines) have been shown to participate in a complex feedback conditioning most of the mitochondrial functions (21), related to health and disease (22).

Globally, these findings suggest differential metabolic sex and aging reprogramming of mitochondria. However, in this analysis other clinical aspects such as BMI or body fat and lifestyle choices have not been considered, and might potentially influence our results. In consequence, further studies should deepen on this topic, considering all potential confounders, as one example of how sex influence may underlie physiologic and physiopathological responses. In this setting, sex differences are frequently ignored in pursuit of simplification and understanding. In accordance with the predominant male perspective, females are usually excluded to meet this aim, leading to biased interpretation of derived conclusions.

Despite past and current literature still exhibits a clear sex bias in clinical and experimental research, there is a growing body of evidence that sexual dimorphism and gender disparity regulate mitochondrial function, metabolism, and, consequently, the response to diet and nutrition. For instance, it has been established that muscle fiber type distribution (greater for type I fibers in females), substrate availability or consumption (higher for lipids and lower for carbohydrates and amino acids in females), as well as ROS production or ADP and oxygen-sensibility (lower in females), are different between sexes (23–28), thus conditioning mitochondrial function and metabolism in physiologic conditions or exercise (glucose turnover, glycogen use, lipid sources, AMPK signaling, lipid droplets metabolism and metabolic gene expression among others) (28, 29). Additionally, the regulation of such metabolic and mitochondrial functions also differs among sexes. For instance, higher levels of circulating adipokines (as adiponectin and leptin) in females, or the presence of 17-β estradiol receptors in muscle (the most important female sex hormone), provide evidence of differential sexual regulation (23), that could vary depending on the menstrual cycle phase (28). Interestingly, such differential sexual metabolic performance in physiologic conditions can constrain the response to disease, for instance in metabolic complications including type 2 diabetes mellitus (30), steatosis or even hepatic failure (31–33), but also in response to exercise (34) or aging (35).

Herein presented data of lower mtDNA content in skeletal muscle of human females, would reinforce the idea of sexual dimorphism in bioenergetic and metabolic interplay, specially studied during exercise (27–29, 33, 36–42), encompassing a wide spectrum of physiologic adaptations, such as those concerning gene expression (38). Moreover, apparent mtDNA decline in females associated with the age of menopause onset may also strengthen estrogen regulation of mitochondrial performance (27–29, 36, 39). Notwithstanding, mtDNA is the unique genome entirely dedicated to metabolic and bioenergetic performance and estrogens, the master regulator of female metabolism and the bioenergetic system (43), thus being the potential base of further adaptations.

Although we have still a long path to explore, these examples provide evidence that we won't understand the basis of health and disease unless sex influence is considered.

## Sex bias in medicine research

In theory, biomedicine and medical research are aimed at understanding health and disease processes, developing new drug therapies, overall, with the objective to reduce the burden of diseases, improve health, and increase lifespan with a minimum quality of life for the overall population. However, in (bio)medicine, most experimental designs (from both cellular and animal models) and a significant amount of patient-derived cohort studies, exclusively included males [reviewed in (44–46)], which account for about only half of the worldwide population (47). This male bias will explain the poor knowledge in the biology and physiology of females. Consequently, several guidelines still do not distinguish differences in the manifestation and treatment of diseases between males and females.

The arguments used to exclude females in its experimental design are diverse and include both objective and subjective approaches. As objective approaches, for instance, the systematic exclusion of females at fertile age to “prevent” them from being submitted to a potentially harmful intervention, or the higher prevalence of a disease in males in a certain range of age. The main subjective approach is the assumption that what is observed in males can be extrapolated to females. The latter two approaches can be easily refuted with the example, for instance, of cardiovascular disease (CVD). CVD is more prevalent in middle-aged male subjects, although overall, CVD kills more females than males, being the first cause of death in females, at least in Europe (48). Moreover, the physiopathology of CVD differs between males and females. Stage 1 diastolic hypertension has been associated with double the risk of an acute coronary syndrome (ACS) in females compared with males (49), and the symptoms of ACS largely differs between sexes, being more often than desirable not identified as ACS symptoms in females (50). Again, this responds to the assumption that the symptoms in females are the same than in males. In addition, the disparities in the prevention, diagnosis and treatment of ACS have been recently brought to light (51), always in detriment of females' health.

Moreover, gender issues such as lifestyle, including nutrition and stress, education and psychological aspects, might influence the outcome of several pathologies. For instance, the prevalence of coronary disease in females is higher and present worst prognosis due to the double exposure to stress from work and family (52). Besides CVD, females have increased risk of experiencing adverse and more severe drugs reactions compared to males (53). A potential explanation of such phenomena might be that female liver cells have increased cytochrome P450 (54), which is the complex responsible of the metabolism of about half of the drugs, thus reducing potential drug's therapeutic efficacy.

In addition, among several others, simplification is another argument used to exclude females from biomedical studies, as half the sample size is then eventually required. In this setting, the hormonal argument (the variability introduced by the menstrual cycle) has been repeatedly used to justify the exclusion of females in (bio)medicine studies. However, it has been largely shown that interindividual variability is usually higher in males (55). Interestingly, males are also subjected to the effects of hormones that vary according to daily and monthly rhythms and, longitudinally, along with their lives (56), and this has not hampered their inclusion in clinical studies. Fortunately, new policies and initiatives such as the Sex As a Biological Variable (SABV) (57), or the GenderMedDB (58), are increasingly being taken into consideration, and within the last years, there is a growing body of research considering both sexes and/or sex differences both in preclinical and clinical studies, which will be further discussed.

## Sex bias in nutritional studies

In nutritional and molecular medicine, sex influence is also frequently underrated, even considering maternal-inherited organelles such as mitochondria. The mitochondrial respiratory chain is the final step for nutrient metabolism and energy production, but sex-differences in nutrient metabolism are largely unexplored, even considering that mitochondrial activity and body fat distribution and percentage largely differs between sexes (59), and that the nutritional requirements differ between males and females (60). Additionally, nutritional requirements within females also differ during pregnancy or after menopause compared to the rest of the adult life. Several current studies which consider the complexity of nutritional and mitochondrial metabolic pathways are unable to consider sex influence on the derived conclusions. Moreover, it has been recently outlined that the micronutrient requirements differ between sexes and across the lifespan (61), although current guidelines do not distinguish between sexes, only having special guidelines for childhood (of both sexes) and pregnancy. Interestingly, a very recent review (62) shows that human breast milk contains lower carbohydrates, lipids and energy for female-term compared to male-term infants, again, showing different sex-associated nutritional requirements even in newborns.

A recent study has analyzed the sex specific effects of a diet-induced obesity, and significant sex-differences in energy expenditure in response to a high-fat diet were found (63). This might have clinical and epidemiological implications, as (severe) obesity is more prevalent in females (64), although a large body of evidence relies on studies performed on males. Moreover, a recent study observed that in children and adolescents with type 1 diabetes, a higher dietary percentage of lipids was more associated with higher levels of LDL cholesterol in girls than in boys (15).

As previously stated, the hormonal argument is recurrent to exclude females in experimental models or cohort studies. This has been the case of the study of the effects of polyphenols, in which, unfortunately, some authors of this perspective have been contributors to this bias in the past (65, 66). Polyphenols are bioactive compounds found in plants with antioxidant and anti-inflammatory activities (67). Given its estrogenic activity (68), it has been argued that the effects of polyphenols may be dependent on the hormonal fluctuations in females, and this may hinder the execution of studies and the interpretation of the results. However, this argument should not be further used, because the effects of nutrients, bioactive compounds, or even dietary patterns might be sexually dimorphic independently of the reproductive hormonal fluctuations in females. A recent study has reported that a dietary pattern rich in energy-dense foods at the age of 4 is associated with a higher body mass index, a higher percentage of body fat, and insulin resistance at the age of 10 in girls, whereas this association was not found in boys (14).

Finally, it is worth mentioning that adherence to dietary patterns is different between males and females (69), which might be also taken into consideration in nutritional epidemiology.

## Conclusion

Cooperation, synergy, and unbiased analysis should be the three pillars in which science should approach any social and health challenge, and sex-biased analyses remains as one of the biggest challenges and limitations to obtaining universal conclusions.

Sex bias in social and historic topics, as well as in medicine and research, can no longer be justified in any circumstance. Females are not a minority, they encompass, at least, half of the human beings. If any bias may be relevant leading to underrating and mistakes, ignoring sex differences in health can cost lives due to the postulation of wrong clinical and therapeutic interventions relying exclusively on man-based studies, thus threatening woman's health and life expectancy.

In this era of OMICs analysis, big data and supercomputational studies, where covariates and confounding factors are included in any calculation, modeling, and conclusion, we can no longer admit studies where sex-based differences are not considered.

In this brief perspective, we have discussed and exposed the sex bias historically inherent to (bio)medical research and nutrition. Considering the genotypic, phenotypic and metabolic biological differences between females and males, and of course, excluding sex-specific research (i.e., pregnancy), sex-biased science should not be acceptable anymore. Therefore, experimental design, at the cellular, animal, and human levels, should include a balanced sex sample, and sex differences might be analyzed and reported for the sake of the overall population.

## Data availability statement

The raw data supporting the conclusions of this article will be made available by the authors, without undue reservation.

## Ethics statement

The studies involving human participants were reviewed and approved by Ethics Committee of the Hospital Clínic de Barcelona. The patients/participants provided their written informed consent to participate in this study.

## Author contributions

GG, FG-G, and GC-B wrote the first draft of the manuscript. All authors contributed to the conception and design of the manuscript, edited the manuscript, suggested improvements at several stages during the preparation, and revised the manuscript.

## Funding

We are grateful for the financial support from the Nutricia Research Foundation and the Spanish Society of Atherosclerosis (a2022-25, and OBN21PE02/2021, respectively, to GC-B). This work was also supported by the Instituto de Salud Carlos III (ISCIII) (PI1800451, PI1800498, and PI2100935 to GG), the Centro de Investigación Biomédica en Red de Enfermedades Raras (CIBERER) (ER20P2AC722 to GG) all initiatives of ISCIII and FEDER (‘Unamanera de hacer Europa'), as well as Fundació Privada Cellex.

## Conflict of interest

The authors declare that the research was conducted in the absence of any commercial or financial relationships that could be construed as a potential conflict of interest.

## Publisher's note

All claims expressed in this article are solely those of the authors and do not necessarily represent those of their affiliated organizations, or those of the publisher, the editors and the reviewers. Any product that may be evaluated in this article, or claim that may be made by its manufacturer, is not guaranteed or endorsed by the publisher.
